# Molecular Significance of Sorbs2 in Alzheimer’s Disease

**DOI:** 10.1002/alz.093433

**Published:** 2025-01-03

**Authors:** Grace Rocco, Henry Lin, Ryan L Boudreau, Jared M McLendon

**Affiliations:** ^1^ University of Iowa, Iowa City, IA USA

## Abstract

**Background:**

Sorbs2 is a cytoskeletal adaptor protein that is expressed in hippocampal neurons, but its mechanistic role in these cells is not yet fully understood.

**Method:**

We created two groups of mice for our study: whole‐body Sorbs2‐Knockout (KO) mice and Sorbs2‐Flox mice, which had neuronal knockout via AAV‐PHP.eB‐hSyn1‐Cre virus injection. We assayed mixed‐sex littermate cohorts at 6 months of age (n = 24 each, WT/KO; n = 8 each ± CRE) with behavior tests. We used postmortem brain samples to visualize and quantify Sorbs2 expression and microtubule stability.

**Result:**

Our preliminary results suggest that female Sorbs2‐KO mice have a decrease in freezing time during contextual fear conditioning at 6 months of age. We next tested an independent cohort of females (n = 8v8) and the data show reduced freezing time in cued fear conditioning and decreased interaction time with the displaced object in Spatial Object Recognition tests after 24 hours. From the above‐mentioned experiments, we have collected tissue samples for molecular and histological analysis. Preliminary results show that global Sorbs2‐KO mice have a complete loss of an immunoreactive band specific to neuronal Sorbs2 isoform at ∼150 kDa, whereas AAV‐Cre injected Sorbs2‐flox mice have > 50% reduction in neuronal Sorbs2. In preliminary studies, we assessed microtubule stability in hippocampus homogenates from global Sorbs2‐KO or WT mice and showed an increase in the amount of free tubulin and a decrease in the amount of polymerized tubulin in the knockout samples. These data suggest that loss of Sorbs2 may destabilize microtubules in the hippocampus.

**Conclusion:**

Our results suggest that Sorbs2 may regulate microtubule stability in the brain and be involved in memory formation or retrieval. In future studies, we plan to identify populations of Sorbs2‐expressing cells in the brain, interrogate neuronal‐specific Sorbs2 deletion, and assess the intersection between Sorbs2 and AD‐related phenotypes in mouse models of neurodegeneration.

